# Complex Regulation of PKCβ2 and PDK-1/AKT by ROCK2 in Diabetic Heart

**DOI:** 10.1371/journal.pone.0086520

**Published:** 2014-01-20

**Authors:** Guorong Lin, Roger W. Brownsey, Kathleen M. MacLeod

**Affiliations:** 1 Molecular and Cellular Pharmacology Research Group, Faculty of Pharmaceutical Sciences, University of British Columbia, Vancouver, Canada; 2 Dept. of Biochemistry and Molecular Biology, University of British Columbia, Vancouver, Canada; UAE University, United Arab Emirates

## Abstract

**Objectives:**

The RhoA/ROCK pathway contributes to diabetic cardiomyopathy in part by promoting the sustained activation of PKCβ2 but the details of their interaction are unclear. The purpose of this study was to investigate if over-activation of ROCK in the diabetic heart leads to direct phosphorylation and activation of PKCβ2, and to determine if their interaction affects PDK-1/Akt signaling.

**Methods:**

Regulation by ROCK of PKCβ2 and related kinases was investigated by Western blotting and co-immunoprecipitation in whole hearts and isolated cardiomyocytes from 12 to 14-week diabetic rats. Direct ROCK2 phosphorylation of PKCβ2 was examined *in vitro*. siRNA silencing was used to confirm role of ROCK2 in PKCβ2 phosphorylation in vascular smooth muscle cells cultured in high glucose. Furthermore, the effect of ROCK inhibition on GLUT4 translocation was determined in isolated cardiomyocytes by confocal microscopy.

**Results:**

Expression of ROCK2 and expression and phosphorylation of PKCβ2 were increased in diabetic hearts. A physical interaction between the two kinases was demonstrated by reciprocal immunoprecipitation, while ROCK2 directly phosphorylated PKCβ2 at T641 *in vitro*. ROCK2 siRNA in vascular smooth muscle cells or inhibition of ROCK in diabetic hearts reduced PKCβ2 T641 phosphorylation, and this was associated with attenuation of PKCβ2 activity. PKCβ2 also formed a complex with PDK-1 and its target AKT, and ROCK inhibition resulted in upregulation of the phosphorylation of PDK-1 and AKT, and increased translocation of glucose transporter 4 (GLUT4) to the plasma membrane in diabetic hearts.

**Conclusion:**

This study demonstrates that over-activation of ROCK2 contributes to diabetic cardiomyopathy by multiple mechanisms, including direct phosphorylation and activation of PKCβ2 and interference with the PDK-1-mediated phosphorylation and activation of AKT and translocation of GLUT4. This suggests that ROCK2 is a critical node in the development of diabetic cardiomyopathy and may be an effective target to improve cardiac function in diabetes.

## Introduction

Diabetic cardiomyopathy is a common complication of diabetes mellitus. The affected hearts initially show diastolic dysfunction, progressing to systolic dysfunction and to the eventual development of left ventricular dilatation and failure [1], one of the most common causes of morbidity and mortality in diabetes. The mechanisms underlying the development of diabetic cardiomyopathy remain to be fully elucidated and no specific treatment is currently available.

At the cellular and molecular levels, hearts from both human patients and animal models of type 1 and type 2 diabetes show changes in energy metabolism, calcium homeostasis, signaling pathways and contractile protein composition [2]. Activation of the RhoA-rho kinase (ROCK) pathway is one such alteration. ROCK is a serine/threonine protein kinase that has two isoforms, known as ROCK1 and ROCK2, which are activated by the small GTP binding protein RhoA. Numerous studies have found increased expression and/or activity of RhoA and ROCK in cardiovascular [3], renal [4], [5] and retinal tissues [6], [7] in animal models of diabetes, while inhibition of ROCK has been found to improve their function [3]–[7]. We have shown that RhoA and ROCK activity are increased in diabetic hearts, and that acute administration of ROCK inhibitors improved their contractile performance [8]. This observation has been lately confirmed in type 1 and type 2 diabetic models undergoing chronic ROCK inhibition [9], [10].

Protein kinase C β2 (PKCβ2) is a member of the conventional subfamily of PKC isozymes [11], [12] and its activation has been found to be a major contributor to diabetic complications in heart [13], [14], vascular [15]–[17], renal and retinal tissues [6], while the PKCβ inhibitors CGP53353 or LY333531 (ruboxistaurin) have been shown to improve the function of heart [13], [14], neural [18], [19] and vascular tissues [16], [17], [20] affected by diabetes. Recently we reported that the RhoA/ROCK pathway contributes to cardiac dysfunction in diabetes at least in part by promoting the sustained activation of PKCβ2 and the production of reactive oxygen species, through a positive feedback loop involving iNOS [21].

The canonical pathway for maturation of conventional PKCs into a catalytically competent form is the sequential phosphorylation of three residues: first, a threonine residue in the activation loop (T500 in PKCβ2) by phosphoinositide-dependent protein kinase-1 (PDK-1), followed by residues in the turn motif (T641) and the hydrophobic motif in the C-terminal region (S660). Although there is evidence that the latter two arise from autophosphorylation, more recently a role for mTORC2 in the phosphorylation of the turn motif in conventional PKCs and in PKCε was demonstrated [22]. In a recent study we found that inhibition of ROCK was associated with attenuation of the increased phosphorylation of PKCβ2 at the T641 site in cardiomyocytes isolated from diabetic but not control hearts [21]. This suggests that under conditions where ROCK expression and activity is increased, it also regulates the phosphorylation of PKCβ2.

The purpose of the present study was to investigate the mechanism of interaction between ROCK and PKCβ2 leading to altered phosphorylation of the latter kinase in diabetic hearts. We found evidence that ROCK2 directly interacts with and phosphorylates PKCβ2 at the T641 site, leading to its activation. Interestingly, inhibition of ROCK-induced PKCβ2 T641 phosphorylation and activity was also associated with increased phosphorylation of PDK-1 and Akt and consequently translocation of GLUT4 to the cell membrane.

## Materials and Methods

### Ethics Statement

All animal study procedures complied with the guidelines of the Canadian Council on Animal Care and all protocols were approved by the Animal Care Committee of the University of British Columbia.

### Induction of Diabetes

Male Wistar rats were treated with streptozotocin (STZ, 60 mg/kg) or its citrate buffer vehicle (0.1 M; pH 4.5) as described [8]. Animals were used for experiments 12–14 weeks after STZ injection; at this time diabetic rats had blood glucose levels of 29.7±1.6 mM (mean ± SE, n = 30) compared to 6.6±0.27 mM (mean ± SE, n = 30) in control rats.

### Extraction of Protein from Rat Cardiac Tissue

Isolated working hearts were perfused with normal buffer or buffer containing 1 µM H-1152 for 30 min as described in our previous study [8]. At this concentration, H-1152 has been shown to maximally inhibit ROCK activity while having no effect on PKC activity in intact tissues [23], [24]. After perfusion, the left ventricle tissues were snap frozen and powdered in liquid nitrogen. The heart tissue (50 mg) was extracted with ice-cold RIPA buffer containing 1x protease and 1x phosphatase inhibitor cocktail. The homogenates were centrifuged at 1000×g for 10 minutes and supernatants were used as total protein. For the membrane fraction, the supernatants were centrifuged at 100,000×g for 60 min, and the pellet was re-suspended in RIPA buffer. The sample protein concentrations were determined by Bradford assay.

### Isolation of Rat Ventricular Cardiomyocytes

Ca^2+^-tolerant cardiomyocytes were isolated from control and diabetic hearts as described previously [8]. Briefly, isolated hearts were perfused in the Langendorff mode with Ca^2+^-free Tyrode’s solution (composition in mM: NaCl 100, KCl 10, KH_2_PO_4_ 1.2, MgSO_4_ 5, taurine 50, glucose 10, HEPES 10), followed by Tyrode’s solution containing 0.05 mM Ca^2+^, 0.8 mg/ml type II collagenase (Worthington Biochem Corp, NJ) and 0.1% BSA. The ventricles were removed, minced, and the resulting cell suspension filtered and centrifuged briefly at 60×g. The cell pellet was washed 3 times in Tyrodes solution containing increasing amounts of Ca^2+^ (0.2 mM, 0.5 mM and 1 mM Ca^2+^). Viability, determined by assessing the percentage of cells that excluded trypan blue dye, ranged from 80% to 90%. The cells were then plated on laminin-coated dishes and cultured in M-199 media for the period of the experiment.

### Western Blotting Analysis and Immunoprecipitation

Western blotting was performed as described previously [8] using the following antibodies: RhoA, ROCK1, ROCK2, PKCα, PKCβ2, PKCε (1∶1000–2000, Santa Cruz Biotechnology Inc, CA), phospho-PKCα T638 and phospho-PKCβ2 S660 (1∶1000, Epitomics, Burlingame, CA), phospho PKCβ1/2 T500 (1∶500, GeneTex, Irvine, CA), AKT (pan), GLUT4, phospho-MYPT1 T696, phospho-AKT S473, phospho-PKCε S729, phospho-PDK-1 S241, phospho-AKT S308, and VAMP2 (1∶1000, Cell Signaling Technology, Boston MA), anti-PDK-1 (1∶500, Genscript, Piscataway, NJ), PHLPP1 and phospho-PKCβ2 T641 (1∶500, Millipore, Lake Placid, NY). Protein band intensity was quantified using Image J software (NIH). For co-immunoprecipitation, equal amounts of protein (500 µg) from control and diabetic hearts were incubated with antibody cross-linked to agarose resin (Pierce Biotechnology), according to the manufacturer’s instructions.

### Smooth Muscle Cell Isolation and Culture

Vascular smooth muscle cells were isolated from the aorta of male Wistar rats by sequential digestion with collagenase I and II and elastase [25]. The cells were maintained in DMEM containing low glucose (5.5 mM) supplemented with 10% fetal calf serum for a maximum of 6 passages. For individual experiments, cells were seeded and grown in culture dishes to near confluence.

### siRNA Transfection

All siRNA and transfection reagents were obtained from Santa Cruz Biotechnology and were used according to the manufacturer’s instructions. Briefly, 2.0×10^5^ vascular smooth muscle cells were seeded in each well of a 6-well culture plate, incubated overnight and transfected the following day. For each transfection, 20 pmols of ROCK2 or scrambled siRNA diluted in 100 µl siRNA transfection medium was mixed with 6 µl of siRNA transfection reagent diluted in 100 µl of siRNA transfection medium at room temperature for 45 minutes to form transfection complexes. The mixture was diluted with 0.8 ml of siRNA transfection medium and was added directly to the cells that had been pre-washed once with 2 ml of siRNA transfection medium. The cells were then incubated for 6 hours. Transfected cells were recovered by adding 1 ml of growth medium with 10% fetal calf serum. After 24 hours, the medium was changed to one containing low glucose (5 mM) or high glucose (20 mM) without serum for another 48 hours. Finally, the medium was removed by aspiration and the transfected cells were washed twice with PBS. SDS gel loading buffer (200 µl) was added to lyse the cells and obtain protein for SDS PAGE and Western blotting.

### In vitro Phosphorylation

PKCβ2 was dephosphorylated with lambda protein phosphatase and then incubated with or without active ROCK2 (Millipore, Lake Placid, NY) in a molar ratio of 1 to 30 in a total volume of 50 µl at 37°C for 45 min. The reaction mixture contained 20 mM tris pH 7.4, 400 µM ATP and 3 mM MgCl_2_. At the end of the incubation period, SDS gel loading buffer (5 µl) was added and the samples were boiled for 5 min. Western blotting was performed with anti-phospho PKCβ Τ500, anti-phospho PKCβ2 Τ641 and anti-phospho PKC S660 antibodies (Cell Signaling Technology Inc, MA).

### ROCK-dependent PKCβ2 Protein Phosphorylation in Diabetic Heart

Total proteins extracted from untreated or ROCK inhibitor-perfused diabetic hearts were immunoprecipitated in RIPA buffer with anti-PKCβ2 antibody coupled to Dynal beads according to the manufacturers’ instruction. The eluted protein was separated by SDS PAGE, followed either by staining with Coomassie Brilliant Blue R250 to demonstrate total PKCβ2 binding proteins or by Western blotting with phospho-(Ser) PKC substrate antibody (Cell Signaling Technology) to identify ROCK-dependent PKCβ2 phosphorylation of proteins.

### Confocal Microscopy

Cardiomyocytes were cultured in M-199 medium on glass bottom culture dishes pre-coated with laminin and allowed to attach for one hour. The medium was then exchanged for fresh M-199 and incubation continued in the presence or absence of ROCK inhibitors for another one hour at 37°C. The cells were washed with ice-cold PBS twice and fixed with 3.7% paraformaldehyde for 20 min. They were permeabilized and blocked with 0.1% NP40 plus 2% BSA in PBS for 45 min at room temperature, then treated with anti-GLUT4 and anti-VAMP2 antibodies (Cell Signaling Technology) at 1∶200 dilution in the same buffer at 4°C overnight. Following three 10 min washes with PBS containing 0.1% NP40 plus 2% BSA, the cells were incubated with a secondary antibody conjugated to Alexa Fluor® 488 or Alexa Fluor® 555 at 1∶1000 for one hour at room temperature. Following another three 10-min washes, the cells were mounted with Prolong Gold Antifade reagents and observed with a Zeiss LSM700 laser scanning microscope.

### In vitro PHLPP Phosphatase Assay

PHLPP is an Mn^2+^-dependent phosphatase and its total activity was determined as described in [26]. The assay was carried out in reaction buffer containing 50 mM Tris (pH 7.4), 1 mM DTT, with or without 5 mM MnCl_2_ at 30°C for 30 min with 5 µg of inactive AKT-1 (Invitrogen) and 50 µg of heart protein extract. Following the incubation, levels of the AKT phosphorylation were analyzed by Western blotting with anti-AKT S473 antibody (Cell Signaling Technology).

### Statistical Analysis

All values are expressed as the mean ± SEM; n denotes the number of animals in each group. Statistical analysis was performed by one-way ANOVA followed by the Newman-Keuls test for multiple comparisons, using GraphPad Prism (GraphPad Software). For all results the level of significance was set at P<0.05.

## Results

### Effect of ROCK Inhibition on the Phosphorylation of PKCβ2

**Figure 1:**
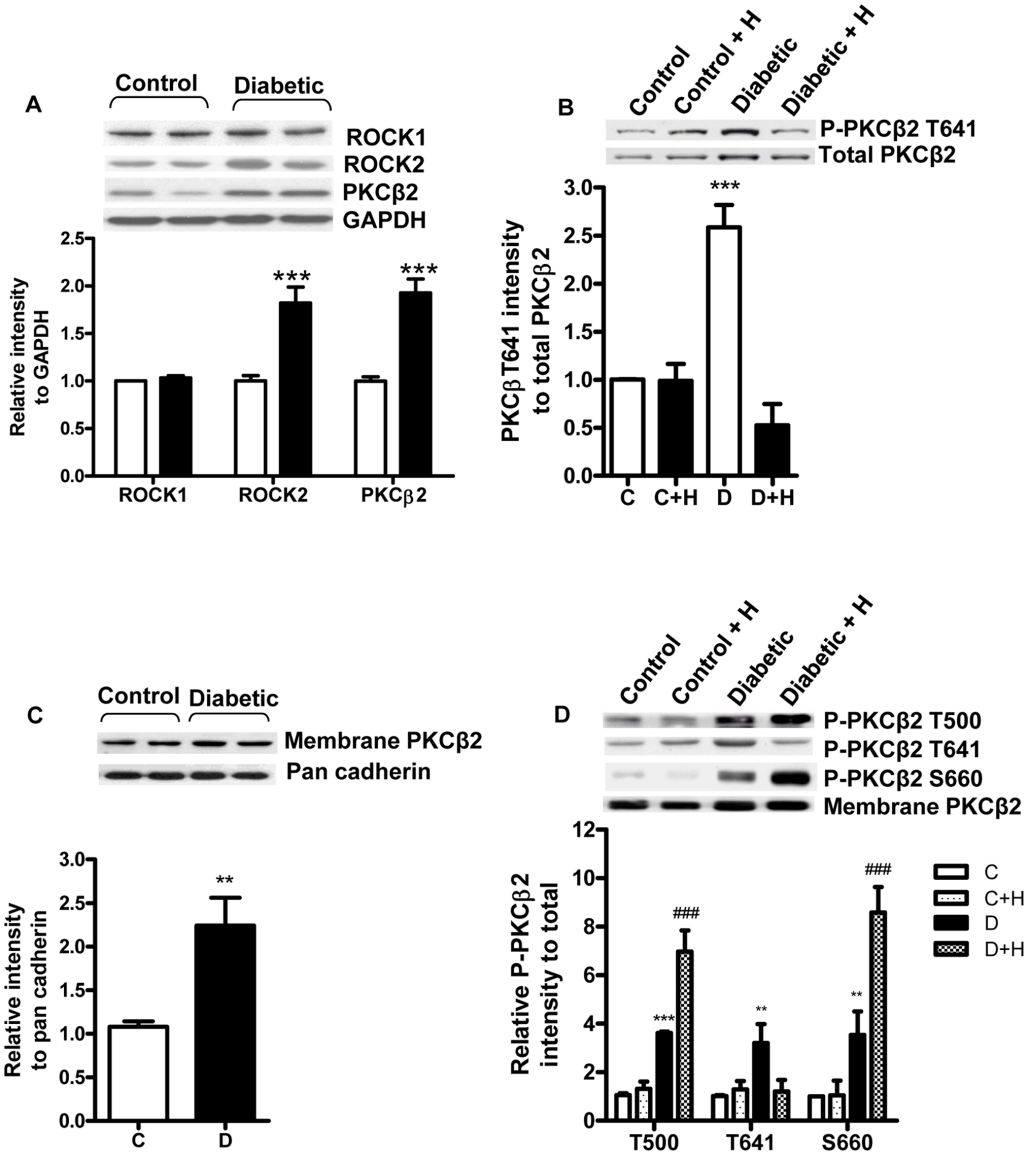
Relationship of ROCK inhibition and PKCβ2 phosphorylation. A. Expression of ROCK1, ROCK2 and total PKCβ2 in left ventricles of control and diabetic hearts. ***, P <0.001 compared to control. B. PKCβ2 T641 phosphorylation in untreated control (C) and diabetic hearts (D), as well as in control and diabetic hearts perfused with 1 µM H-1152 (C+H, D+ H). ***, P <0.001 compared to all groups. C. Levels of total PKCβ2 in the membrane fraction of untreated control (C) and diabetic (D) hearts; **, P<0.01 compared to control. D. Levels of phosphorylated PKCβ2 relative to total in the membrane fraction of untreated (C, D) and ROCK inhibitor (C+H, D+ H; 1 µM H-1152) treated hearts. ***, P<0.001 compared to corresponding untreated control; **, P<0.01 compared to all other groups; ###, P<0.001 compared to all other groups.

We have previously shown that ROCK activity is increased in hearts from diabetic rats, and that its acute inhibition improves contractile function [8]. Further investigation of these hearts revealed that the expression of both ROCK2 and PKCβ2 was increased while ROCK1 levels were not changed in diabetic hearts compared to control (fig. 1A). The phosphorylation of PKCβ2 T641 was increased to a relatively greater extent than its total expression in diabetic hearts, but the ratio of phospho-T641 to total PKCβ2 was normalized after acute perfusion of diabetic hearts with the ROCK inhibitor H-1152 (fig. 1B). In contrast, inhibition of ROCK had no effect on the phosphorylation of either PKCα or PKCε isofoms at the sites corresponding to T641 (T638 in PKCα and S729 in PKCε; data not shown). Since PKCβ2 activation is associated with its translocation to the plasma membrane, we next determined levels of total and phosphorylated PKCβ2 in the membrane fraction. Total PKCβ2 levels were increased in the membrane fraction of untreated diabetic hearts compared to control, consistent with its increased activation (fig. 1C). In addition, there was increased phosphorylation of PKCβ2 at all three residues. As expected, acute perfusion with the ROCK inhibitor H-1152 normalized the phosphorylation of T641 in the membrane fraction of diabetic hearts. However, the phosphorylation of the T500 and S660 sites was further increased in the presence of H-1152 (fig. 1D).

**Figure 2:**
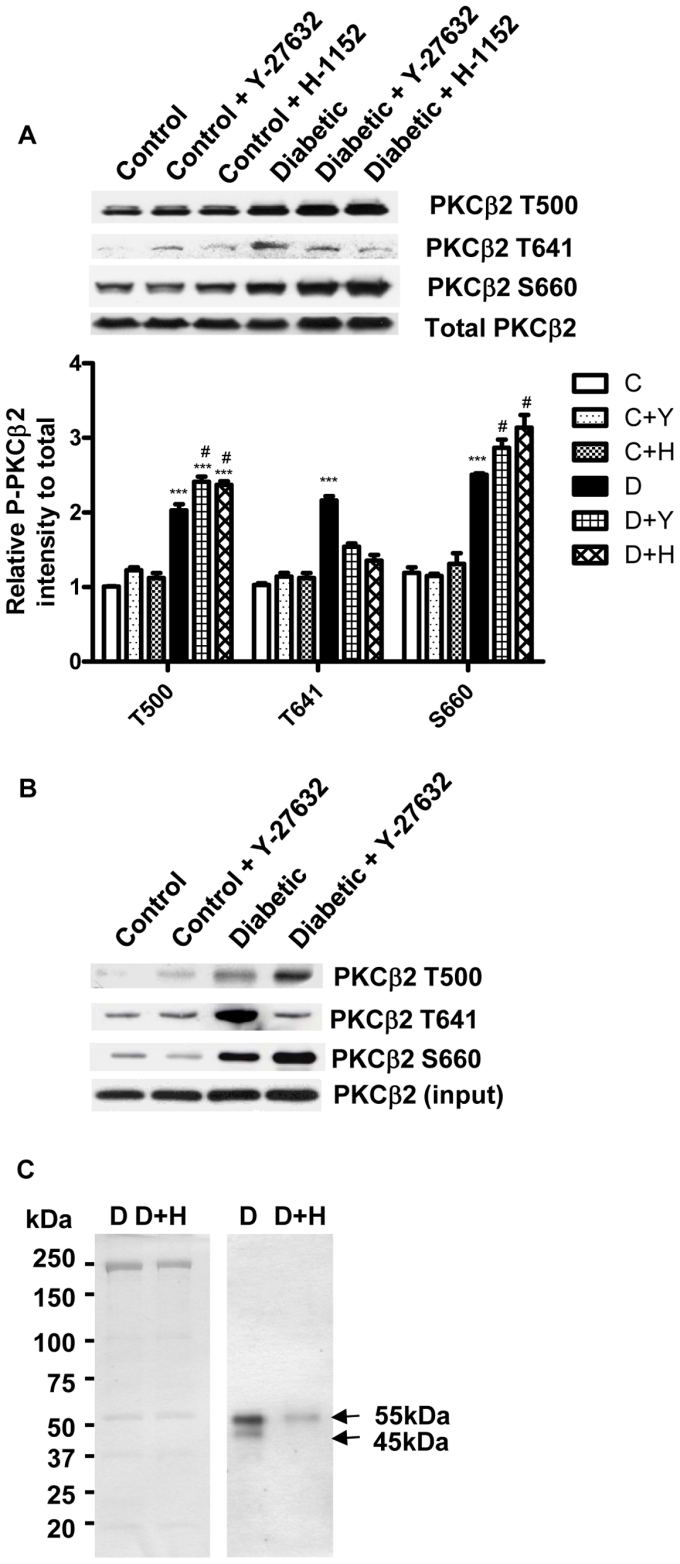
Effect of ROCK inhibition on phosphorylation of PKCβ2 and its targets. A. Phosphorylation of PKCβ2 in cardiomyocytes isolated from control (C) and diabetic (D) hearts. Cardiomyocytes were either untreated (C,D) or incubated with 1 µM Y-27632 (C+Y, D+Y) or 1 µM H-1152 (C+H, D+H). ***, P<0.001 compared to corresponding control groups; #, P<0.05 compared to corresponding untreated diabetic group, n = 4 in each group. B. Representive immunoblot showing phosphorylation of PKCβ2 immunoprecipitated from untreated and Y-27632-treated control and diabetic hearts. C. Co-immunoprecipitation of PKCβ2 binding proteins with anti-PKCβ2 antibody coupled to Dynal beads, followed by detection by Coomassie Brilliant Blue staining (left). ROCK-dependent PKCβ2 phosphorylation targets were detected by anti-PKC phospho substrate antibody (right). Arrows indicate the two protein bands as PKCβ2 downstream targets.

In order to confirm this unexpected observation we investigated the effects of H-1152 and those of a second, chemically distinct, ROCK inhibitor, Y-27632, on the phosphorylation of PKCβ2 in cardiomyocytes isolated from control and diabetic hearts (fig. 2A). As was found in whole heart, total levels of PKCβ2 and the phosphorylation of all three residues were increased in untreated cardiomyocytes from diabetic compared to control hearts. Neither H-1152 nor Y-27632 had any effect on the phosphorylation of PKC in control cardiomyocytes. However, the phosphorylation of T641 was completely abrogated, while that of T500 and S660 was significantly increased in the presence of both inhibitors in cardiomyocytes from diabetic rat heart (fig. 2A).

Because of similarities between the different PKC isoforms in the sequences of the activation loop, hydrophobic motif and turn motif, anti-phospho PKCβ2 antibodies may cross-react with the same sites in other isoforms. In order to rule out a contribution of other isoforms to the changes in phosphorylation detected, we also determined phosphorylation of the T500, T641 and S660 residues in PKCβ2 immunoprecipitated from diabetic hearts (fig. 2B). The results were similar to those obtained in tissue extracts, and demonstrated that the phosphorylation of all 3 residues was increased in untreated diabetic hearts, and that T641 phosphorylation was reduced, while T500 and S660 phosphorylation was increased in the presence of Y-27632 (fig. 2B).

Taken together, these data suggest that ROCK regulates the phosphorylation of PKCβ2 T641 in diabetic hearts, but that the phosphorylation of the T500 and S660 residues is controlled by a different mechanism, which is examined further below.

### Effect of ROCK Inhibition on PKCβ2 Activity

Since the phosphorylation of T641 is an absolute requirement for PKCβ2 activity [27]–[29], the decrease in its phosphorylation on inhibition of ROCK implies that its activity was also reduced in diabetic hearts. However, no PKCβ2-specific phosphorylation targets have been conclusively identified in the heart with which to confirm this possibility. To overcome this issue, we isolated PKCβ2 binding proteins by co-immunoprecipitation with an anti-PKCβ2 antibody, followed by detection with an anti-phospho-(Ser) PKC substrate antibody to detect those whose phosphorylation was sensitive to ROCK inhibition. A number of protein bands were detected by Coomassie Brilliant Blue following co-immunoprecipitation with the anti-PKCβ2 antibody, but only two bands, in the region of 45 kDa and 55 kDa showed significant levels of phosphorylation (fig. 2C). Inhibition of ROCK appeared to have no effect on total protein isolated, but greatly reduced the phosphorylation of the 55 kDa band and completely eliminated phosphorylation of the 45 kDa band (fig. 2C) confirming the reduction of PKCβ2 activity by ROCK inhibition.

### Evidence of Physical Interaction of ROCK2 and PKCβ2

**Figure 3:**
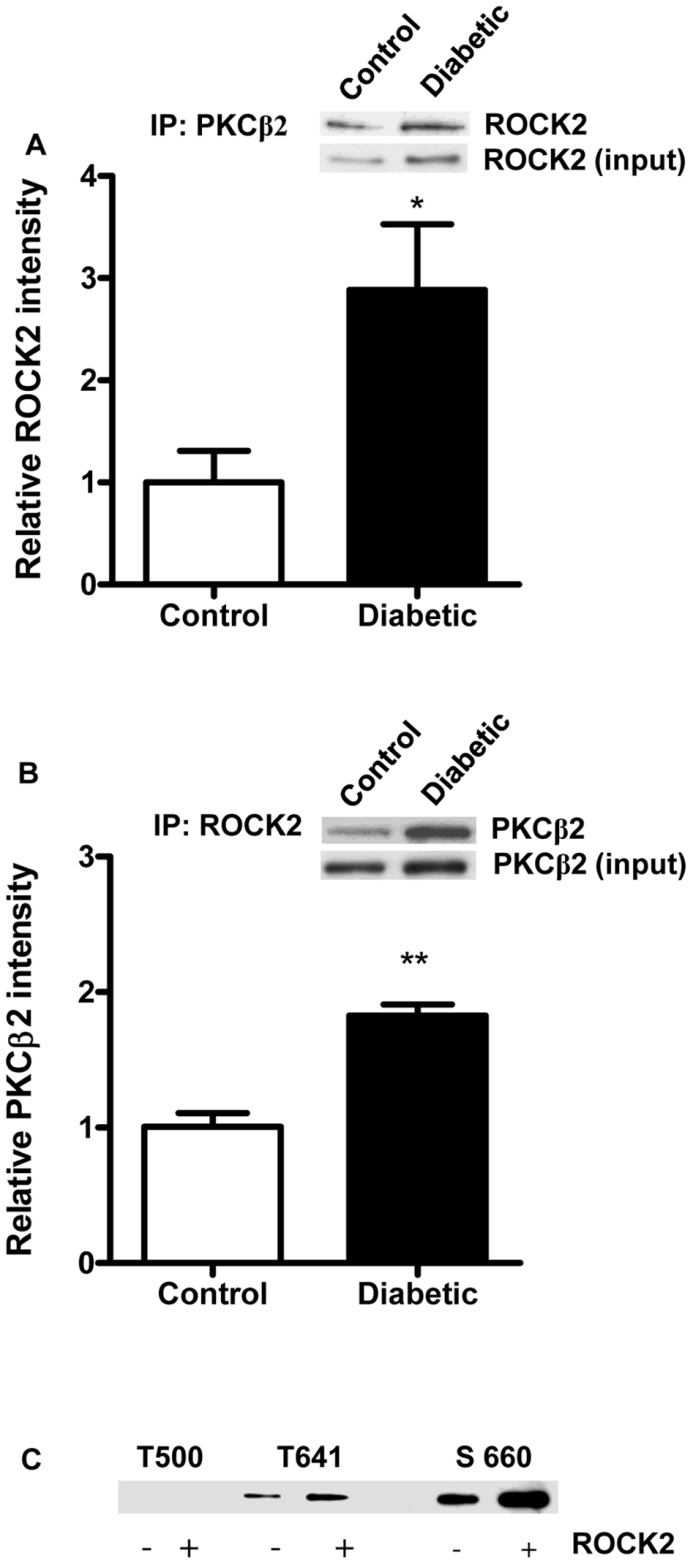
Interaction of ROCK2 and PKCβ2 in control and diabetic hearts. A. Representive immunoblot (top) and quantification (below) of ROCK2 from control and diabetic hearts immunoprecipitated (IP) with anti-PKCβ2 antibody. n = 3; *P<0.05 compared to control. B. Representive immunoblot (top) and quantification (below) PKCβ2 from control and diabetic hearts immunoprecipitated (IP) with anti-ROCK2 antibody. n = 3; **P<0.01 compared to control. C. *In vitro* phosphorylation of PKCβ2 by ROCK2. The ROCK2 to PKCβ2 molar ratio was 1∶30 in the *in vitro* phosphorylation assay. The specific phosphorylation sites were detected with the correspond antibodies indicated.+sign indicates addition of ROCK2 in the assay.

In order to determine whether there was a physical association between ROCK2 and PKCβ2 in the heart, we conducted reciprocal immunoprecipitation with anti-ROCK2 and anti-PKCβ2 antibodies. The results show that ROCK2 was immunoprecipitated from both control and diabetic hearts by anti-PKCβ2 antibody (fig. 3A) and similarly that PKCβ2 was immunoprecipitated from both control and diabetic hearts by anti-ROCK2 antibody (fig. 3B). However, as expected, greater amounts of both kinases were immunoprecipitated from diabetic heart (fig. 3A and 3B).

To determine whether ROCK2 is able to directly phosphorylate PKCβ2, we carried out an *in vitro* phosphorylation reaction in the presence of ATP. The results showed that levels of PKCβ2 T641 and S660 phosphorylation were increased by approximately 2 and 2.5 fold respectively in the presence compared to the absence of ROCK2. However, no phosphorylation of the T500 residue could be detected (fig. 3C).

### Effect of ROCK2 siRNA on the Phosphorylation of PKCβ2 T641

**Figure 4:**
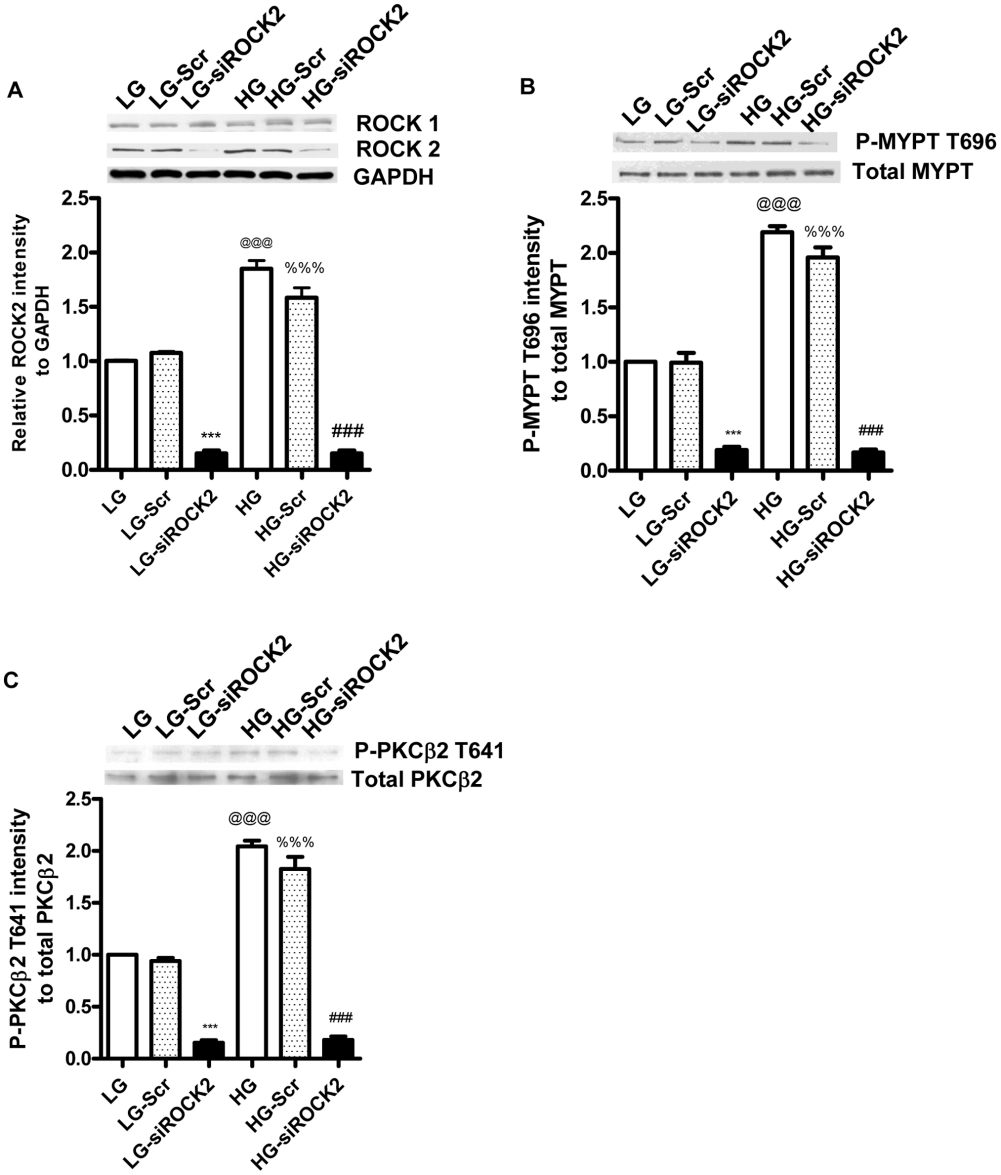
ROCK2 siRNA reduces PKCβ2 T641 phosphorylation in cultured vascular smooth muscle cells. Vascular smooth muscle cells were transfected with scrambled siRNA (Scr) or ROCK2 siRNA (ROCK2) for 24 hours, following which the medium was removed and changed to one containing 5 mM glucose (LG) or 20 mM glucose (HG) for an additional 48 hours. Levels of ROCK1 and ROCK2 (A), total MYPT and phospho-MYPT T696 (B) as well as total and phospho-T641 PKCβ2 (C) were determined by Western blot. *** P<0.001 to all others in the same group; @@@ and %%% P<0.001 to the corresponding group cultured in low glucose by one-way ANOVA.

The above results suggest that ROCK2 may be the ROCK isoform increasing the phosphorylation of PKCβ2 at the T641 site in diabetic hearts. We have previously demonstrated that similar changes in phosphorylation of PKCβ2 can be detected in cardiomyocytes and blood vessels from diabetic rats and in both adult cardiomyocytes and vascular smooth muscle cells cultured in high glucose [20]. Therefore, we used vascular smooth muscle cells cultured in high glucose to investigate the effect of ROCK2 knockout on phosphorylation of PKCβ2 T641 using siRNA gene silencing, an approach which is not feasible in adult cardiomyocytes, particularly those isolated from diabetic hearts [21]. We first demonstrated that the expression of ROCK2 but not ROCK1 was increased in vascular smooth muscle cells incubated in high glucose (fig. 4A) and that this was associated with an increase in ROCK activity (fig. 4B) and in the phosphorylation of PKCβ2 at the T641 site (fig. 4C). As observed in diabetic hearts, the increased phosphorylation of PKCβ2 T641 was sensitive to inhibition of ROCK (data not shown). ROCK2 siRNA reduced protein levels of ROCK2 by more than 90% in vascular smooth muscle cells incubated either low or high glucose while the expression of ROCK1 was not affected (fig. 4A). The reduced expression of ROCK2 was associated with a parallel reduction in the phosphorylation of MYPT T696 and PKCβ2 at the T641 site, although total levels of PKCβ2 remained unchanged (fig. 4B and 4C).

### Effect of Diabetes and ROCK Inhibition on PDK1 and PHLPP Activity

**Figure 5:**
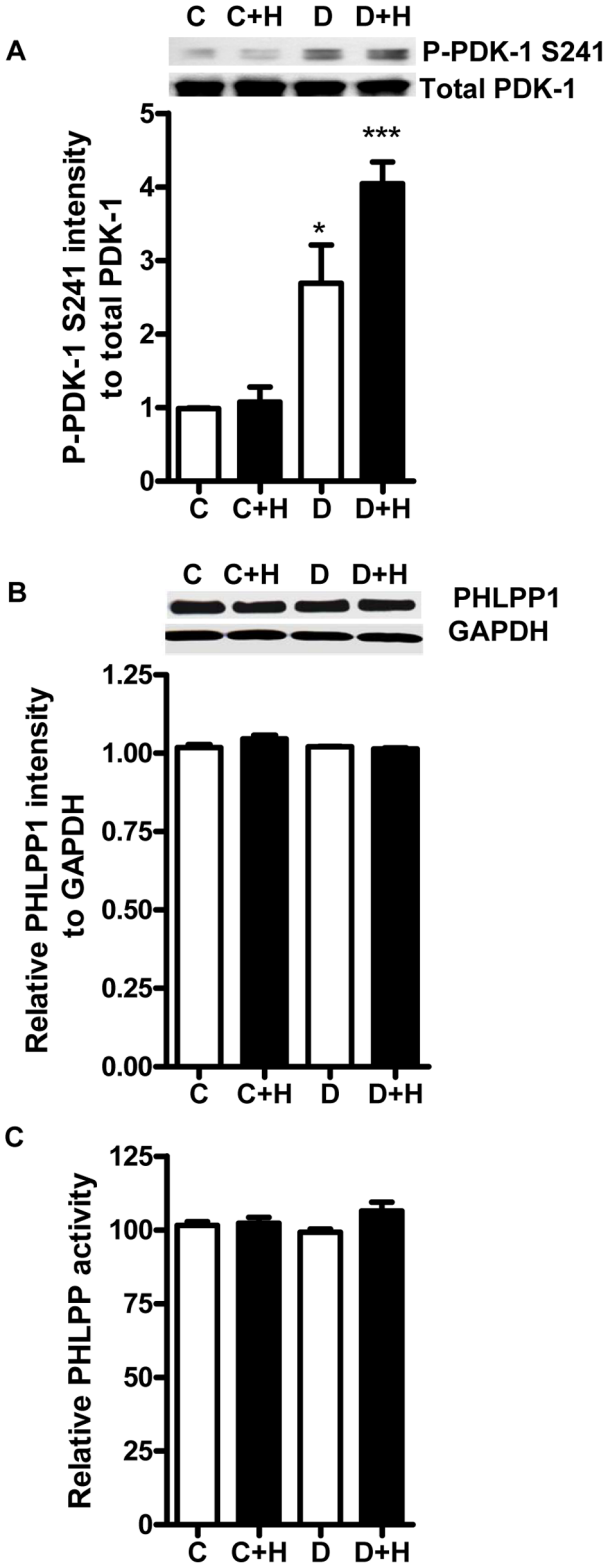
Effect of diabetes and ROCK inhibition on PDK-1 and PHLPP. Levels of total and phospho-PDK-1 (A), expression of PHLPP1 (B) and total PHLPP activity (C) in untreated control (C) and diabetic hearts (D), as well as in control and diabetic hearts perfused with 1 µM H-1152 for 30 min (C+H, D+ H). *, P<0.05 compared to corresponding untreated control; **, P<0.01 compared to all other groups, n = 4 in each group.

We next investigated the basis for the change in phosphorylation of PKCβ2 T500 and S660 in diabetic hearts in the presence and absence of ROCK inhibition. PDK-1 is activated by autophosphorylation of S241 in the activation loop [30], [31] and is generally considered to be the kinase responsible for the phosphorylation of T500 in PKCβ2. Comparison of control and diabetic hearts showed that the expression of PDK-1 was not affected by diabetes or by ROCK inhibitor treatment. However, the phosphorylation of PDK-1 at the S241 site, which often appears as double bands of different phosphorylation forms [32], was significantly increased in untreated diabetic hearts and was further enhanced by inhibition of ROCK, suggesting that it’s activity was increased under both circumstances (fig. 5A). These changes correlate very well with the changes in phosphorylation of PKCβ2 T500 and S660 in the diabetic heart.

PH domain leucine-rich repeat protein phosphatases (mainly PHLPP1 and PHLPP2 isoforms) have been shown to dephosphorylate PKCβ2 and other PKCs [26]. However, no changes in the expression of PHLPP1 (fig. 5B) or in total PHLPP activity (fig. 5C) were detected in diabetic hearts in either the presence or absence of ROCK inhibitor.

### Effect of Diabetes and ROCK Inhibition on PDK-1 Induced AKT Phosphorylation and Translocation of GLUT4

**Figure 6:**
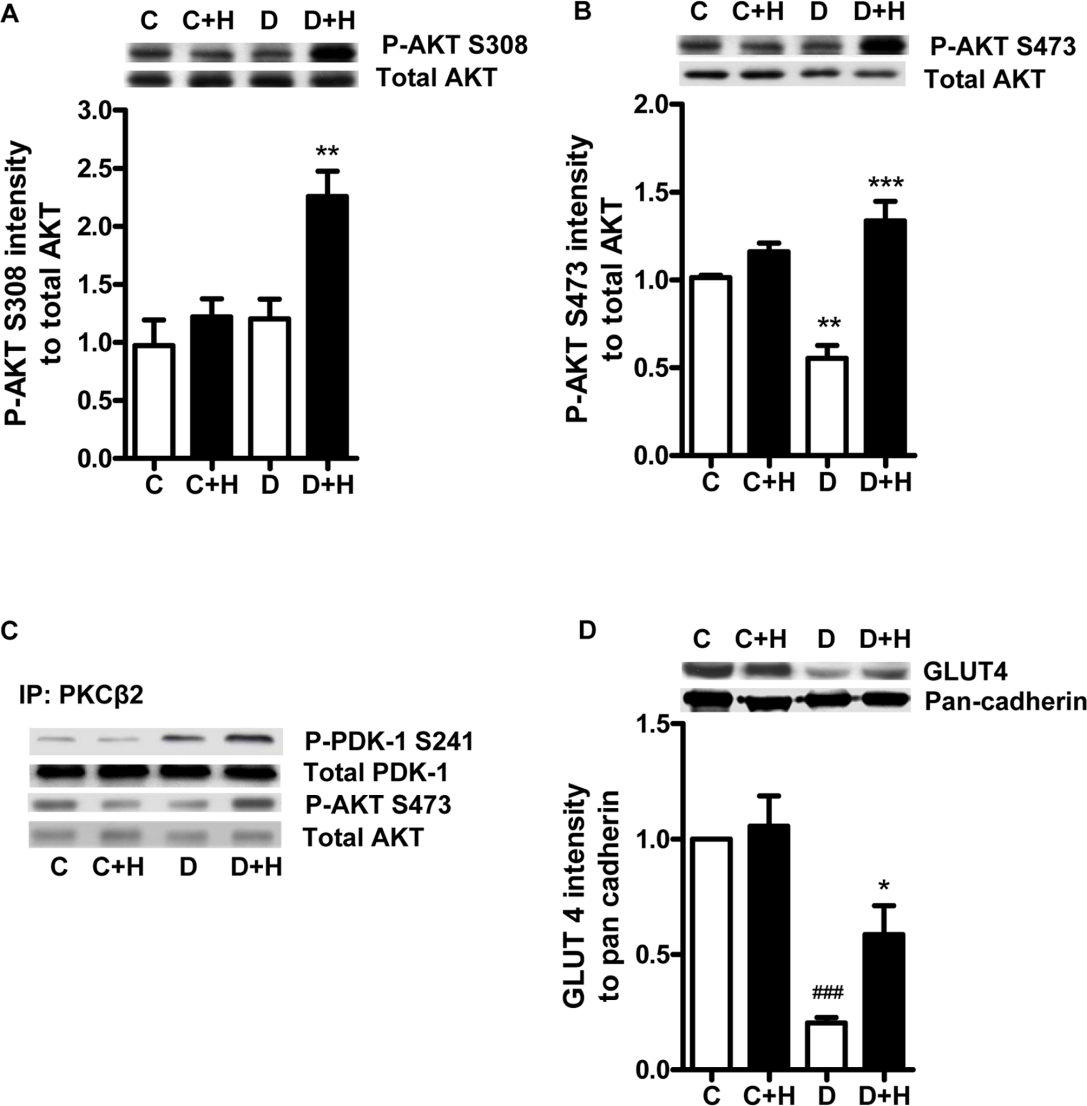
Effect of ROCK inhibition on PDK-1 and AKT- 1 signalling and GLUT4 translocation. A. Levels of total and phospho-S308 AKT in untreated (C, D) and ROCK inhibitor (C+H, D+ H; 1 µM H-1152) treated hearts. **,P<0.01 compared to all other groups; B. Levels of total and phospho-S473 AKT in untreated (C, D) and ROCK inhibitor (C+H, D+ H; 1 µM H-1152) treated hearts. **,P<0.01, ***P<0.001 compared to all other groups. C. Representative Western blot of total and phospho- PDK-1 and AKT from untreated (C, D) and ROCK inhibitor (C+H, D+ H; 1 µM H-1152) treated hearts co-immunoprecipitated (IP) with anti-PKCβ2 antibody. D. Levels of GLUT4 in the membrane fraction of untreated (C, D) and ROCK inhibitor (C+H, D+ H; 1 µM H-1152) treated hearts. *,P<0.05 compared to corresponding untreated diabetic.

AKT is an immediate downstream target of PDK-1, so we determined whether phosphorylation of this kinase was altered in diabetic compared to control hearts. In untreated diabetic hearts, T308 phosphorylation in the AKT activation loop, which is the PDK1 site, was unchanged (fig. 6A) while phosphorylation of S473 at the hydrophobic motif was reduced compared to control (fig. 6B). However, following perfusion of diabetic hearts with ROCK inhibitor, AKT T308 phosphorylation was significantly increased compared to both control and untreated diabetic hearts, and this was associated with normalization of phosphorylation at the S473 site (fig. 6A and B).

Both PDK-1 and Akt co-immunoprecipitated with the anti-PKCβ2 antibody, suggesting that these proteins form a signaling complex with PKCβ2 in cardiomyocytes (fig. 6C). Total PDK-1 binding to PKCβ2 was similar in control and diabetic hearts and was not affected by ROCK inhibition (fig. 6C). However, as was found in whole heart, the S241 phosphorylation of PDK1 was increased in immunoprecipitates from untreated diabetic hearts, and was further increased following inhibition of ROCK. Similarly, no change in total levels of AKT interacting with PKCβ2 was found in untreated or ROCK inhibitor treated diabetic hearts compared to control, although phosphorylation at the S473 site was reduced in untreated diabetic hearts and restored following ROCK inhibition (fig. 6C).

**Figure 7:**
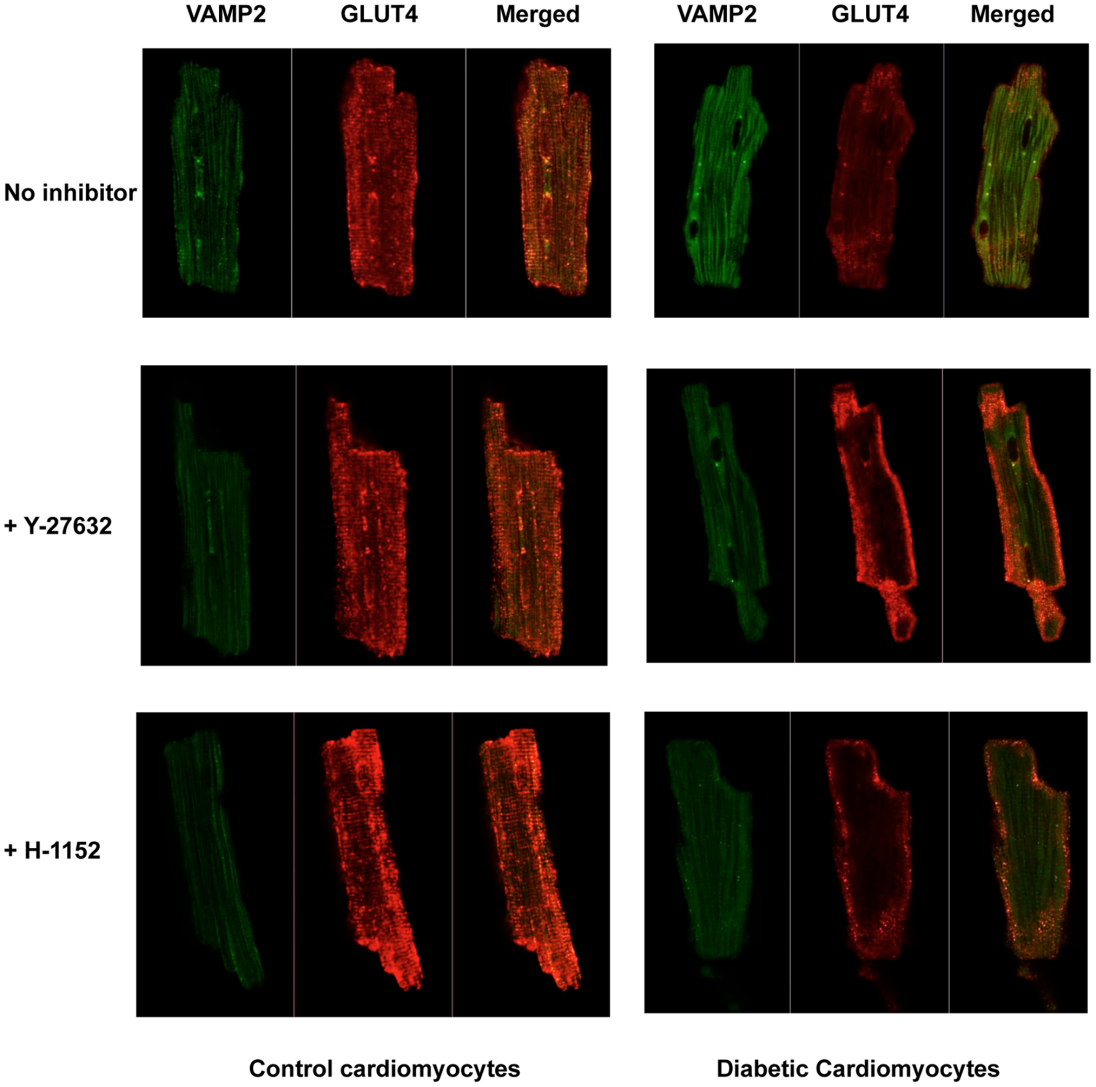
Confocal microscopy of GLUT4 and VAMP2 co-localization in control and diabetic cardiomyocytes. Cardiomyocytes from control and diabetic hearts were treated with either 1 µM Y-27632 or 1 µM H-1152 for one hour or remained untreated, and the localizations of VAMP2 (green, Alexa Fluor® 488) and GLUT4 (red, Alexa Fluor® 555) were determined by Zeiss LSM 700 laser scanning microscope. The images were analyzed with Zen software to show on their co-localization (merged).

Since AKT regulates the translocation of GLUT4 to the membrane, we investigated whether the levels of GLUT4 in the membrane fraction were affected by ROCK inhibition. As reported previously [33], GLUT4 levels were much lower in the membrane fraction of untreated diabetic than control hearts. Treatment with the ROCK inhibitor H-1152 for 30 min had no effect on GLUT4 translocation in control hearts, but partially restored GLUT4 translocation to the plasma membrane in diabetic hearts (fig. 6D). Consistent with this, confocal analysis of cardiomyocytes isolated from control and diabetic hearts revealed that levels of GLUT4 associated with the plasma membrane were much lower in diabetic than control cardiomyocytes, and that very little co-localization of GLUT4 with VAMP2 (vesicle-associated membrane protein 2) could be detected (fig. 7). VAMP2 has been shown to be essential for GLUT4 translocation and fusion with the plasma membrane [34]. Incubation of cardiomyocytes from control hearts with either H-1152 or Y-27632 had no effect on GLUT4 expression or its co-localization with VAMP2, but both inhibitors increased levels of both GLUT4 and VAMP2 at the plasma membrane of diabetic cardiomyocytes (fig. 7).

## Discussion

Both acute and chronic inhibition of the RhoA-ROCK pathway improve the contractile function of the diabetic heart [8]–[10], and we have previously demonstrated that this results at least in part from decreased activation of PKCβ2 [21]. The novel findings of the present study are that: 1) ROCK2 but not ROCK1 expression is selectively upregulated in diabetic hearts; 2) ROCK2 directly interacts with and phosphorylates PKCβ2 at the T641 site; and 3) ROCK inhibition in cardiomyocytes not only blocked the activation of PKCβ2 but also induced the activation of PDK-1/Akt leading to increased GLUT4 translocation. These data suggest that increased expression and activity of ROCK2 in the diabetic heart contributes to diabetic cardiomyopathy by modifying the activity of multiple signaling pathways.

ROCK1 and ROCK2 have a molecular weight of approximately 160 kDa and share up to 65% overall homology with 92% homology in the kinase domain [35], but some of their targets and functions are distinct. For example, although both ROCK1 and ROCK2 promote the phosphorylation of MLC and MYPT in vascular smooth muscle cells, silencing of ROCK2 was found to lead to a greater reduction in force production than silencing of ROCK1 [36]. On the other hand, ROCK1 was shown to be required for insulin-induced GLUT4 translocation in cultured cells, and targeted knockout of ROCK1 caused whole body insulin resistance in mice [37], [38]. In contrast, we found that expression of ROCK2 is selectively increased in diabetic hearts, and that this isoform is responsible for the increased phosphorylation of PKCβ2 at the T641 site. These data suggest that ROCK2 has a unique role in diabetic cardiomyopathy to regulate the activity of PKCβ2.

The phosphorylation of PKCβ2 at each of the three sites essential for its full activation was uniformly increased in diabetic hearts, although through distinct mechanisms. Previous studies have suggested that mTORC2 is important in the phosphorylation of the turn motif, the site most critical for kinase activity, in conventional PKCs including PKCβ2 [22], [39]. However, a number of observations are consistent with the possibility that in the diabetic heart, ROCK2 directly interacts with and phosphorylates PKCβ2 at T641. First, both ROCK2 and PKCβ2 could be reciprocally co-immunoprecipitated with their counterpart antibodies. In addition, ROCK2 was able to directly increase the phosphorylation of PKCβ2 T641 *in vitro*. Finally, the selective silencing of ROCK2 profoundly attenuated PKCβ2 phosphorylation at the T641 site in vascular smooth muscle cells. On the other hand, a role of mTORC2 in this process seems unlikely, since the increased phosphorylation of PKCβ2 in the diabetic heart was not accompanied by a corresponding increase in the phosphorylation of PKCα at the turn motif, while the phosphorylation of AKT at the S473 site, a well-established mTORC2 target used to assess mTORC2 activity [22], was actually reduced in untreated diabetic hearts.

PHLPP1 and PHLPP2 are the phosphatases that are believed to control the intracellular levels of PKCβ2 through dephosphorylation of T641 and S660, resulting in degradation of the kinase [26], and a decrease in their activity could also result in increased phosphorylation of PKCβ2. However, in the present study the total activity of PHLPP was not changed in untreated diabetic hearts in comparison to control. Furthermore, inhibition of ROCK, which had no effect on the activity of PHLPP, was associated with a further increase in the phosphorylation of S660, while removal of the phosphate from this residue has been shown the rate-limiting step in the PHLPP-mediated dephosphorylation of PKCβ2 [26]. We have previously reported that the activity of other phosphatases that have been shown to dephosphorylate PKC isoforms in vitro, including PP1 and PP2A, is not altered in diabetic hearts [40]. Based on these results, it seems unlikely that the increased T641 PKCβ2 phosphorylation in diabetic hearts is due to a decrease in phosphatase activity.

ROCK2 had no effect on the phosphorylation of PKCβ2 at T500 site in the *in vitro* phosphorylation assay, although phosphorylation of S660 was increased, likely through autophosphorylation. PDK-1 has been demonstrated to be the kinase responsible for the activation loop phosphorylation in PKCβ2 as well as other PKC isoforms [41], [42], and the increased phosphorylation of PKCβ2 at the T500 site in diabetic hearts was associated with an increase in the activity of PDK-1, indicated by its phosphorylation at S241. Furthermore, treatment of diabetic hearts with ROCK inhibitor further increased the phosphorylation of PDK-1 S241, while at the same time the phosphorylation levels of PKCβ2 T500 and S660 were increased in parallel. These data suggest that PDK-1 is responsible for the enhanced phosphorylation of T500 in the diabetic heart. The associated increase in phosphorylation of S660 may result from autophosphorylation, since the hydrophobic motif in PKCα is subject to autophosphorylation secondary to increased phosphorylation of either the activation loop or the turn motif [22]. The mechanism responsible for the increase in PDK-1 S241 phosphorylation in the diabetic heart and its further increase following ROCK inhibition is unclear and remains to be further investigated.

**Figure 8:**
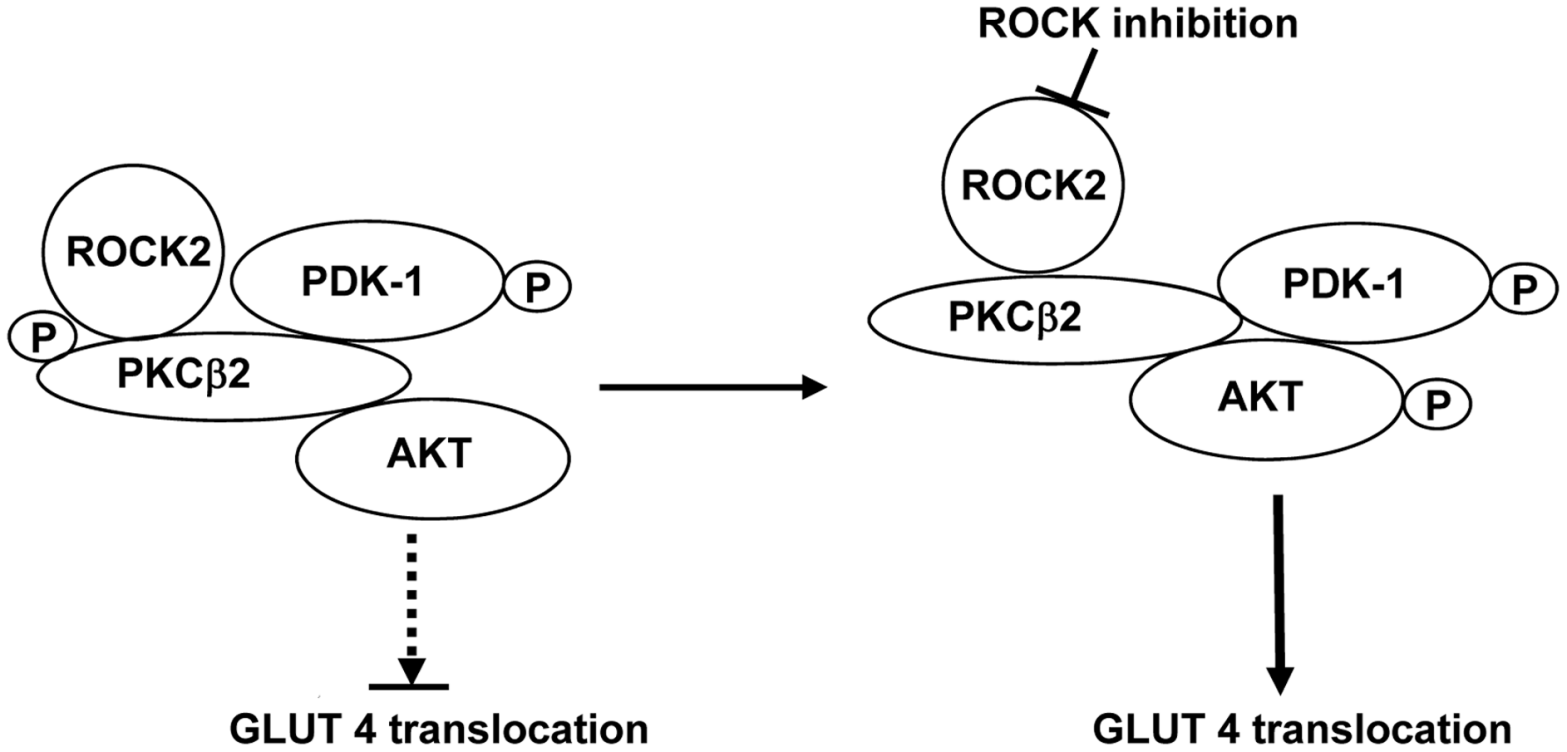
Model of kinase interactions and their effects on GLUT4 translocation. ROCK2, PKCβ2, PDK-1 and AKT may all form part of a signaling complex. When ROCK2 activity and PKCβ2 T641 phosphorylation are elevated in untreated diabetic hearts, the interaction of PDK-1 with AKT, but not PKCβ2 itself is prevented. However, on inhibition of ROCK2, the associated decrease in PKCβ2 T641 phosphorylation appears to promote the phosphorylation by PDK-1 of both PKCβ2 and AKT, resulting in phosphorylation and activation of the latter. The resulting increase in activation of AKT may then increase GLUT4 translocation to the membrane.

Akt is an important signaling hub that regulates cell metabolism, proliferation and survival [43]. Phosphorylation of both T308 in the activation loop by PDK1 and of S473 at the hydrophobic motif in the carboxy terminus by mTORC2 is required for its full activation [44]. Expression of constitutively active Akt in the heart has been shown to increase GLUT4 expression in sarcolemmal membranes and to enhance glucose uptake in cultured cells [45]. The interaction of PDK1 with Akt is known to be very different from that of PDK1 and other kinases such as PKCβ2. Both PDK-1 and Akt have a pleckstrin homology (PH) domain that binds to phosphoinositide for their recruitment to the cell membrane [46], [47]. The two proteins are able to form a complex in the cytosol in the basal state, but T308 phosphorylation cannot take place because of an interaction between the PH and kinase domains of Akt [47]. The binding to phosphoinositide induces a conformation change in Akt, which allows PDK-1 access to phosphorylate T308 [48]. In contrast, the interaction of PDK1 with PKCβ2 involves its interaction with the carboxy terminus of the latter, and is independent of the PH domain [49]. In the present study, although both PDK-1 and AKT co-immunoprecipitated with PKCβ2, and PDK-1 S241 phosphorylation was increased, we found no change in the phosphorylation of AKT at T308 in untreated diabetic hearts. However, following treatment of diabetic hearts with ROCK inhibitor, the further increase in phospho-S241 PDK-1 levels was associated with a marked increase in AKT phosphorylation at T308 and normalization of phosphorylation of the S473 site. Taken together, our results are consistent with a model in which ROCK2, PKCβ2, PDK-1 and AKT all form part of a signaling complex. When ROCK2 and PKCβ2 activity are elevated in untreated diabetic hearts, the ability of PDK-1 to phosphorylate Akt, but not PKCβ2, is restricted. However, on inhibition of ROCK2, the associated decrease in PKCβ2 T641 phosphorylation and activity appears to allow the increased phosphorylation of T308 by PDK-1. At the same time, phosphorylation of S473-Akt is normalized, as was recently found following direct inhibition of PKCβ in diabetic hearts [14]. This leads to increased AKT downstream signaling and promotion of GLUT4 translocation to the plasma membrane (fig. 8).

ROCK2 siRNA was much more effective than the pharmacological inhibition of ROCK in reducing PKCβ2 T641 phosphorylation in vascular smooth muscle cells incubated in high glucose. Furthermore, ROCK2 silencing also reduced PKCβ2 T641 phosphorylation in normal glucose, although pharmacological inhibition of ROCK had no detectable effect on this parameter. These results may be explained by the observation that proteins treated with small molecule inhibitors may still act as a scaffold for protein-protein interactions that are disrupted by siRNA treatment [50]. It is possible that ROCK2 acts as a scaffold for PKCβ2 and its associated proteins even under normoglycemic conditions when its activity is low. This is supported by our observations that that the two kinases co-immunoprecipitated with their corresponding antibodies from non-diabetic hearts. The disruption of the ROCK2-PKCβ2 interaction resulting from the siRNA-induced decrease in ROCK2 levels may either attenuate PKCβ2 phosphorylation or allow its effective dephosphorylation under these circumstances.

Inhibition of ROCK leads to greatly reduce PKCβ2 activity as showed the diminution of phosphorylation in 55 and 45 kDa protein bands. Further studies are underway to identify the ROCK2-dependent PKCβ2 targets and determine whether they are new targets contributing to dysfunction in diabetic heart. Importantly, it will be of significance to determine whether ROCK2, PKCβ2, PDK-1 and AKT also form a signaling complex as a mechanism to regulate GLUT4 translocation in adipose tissues and skeletal muscle in type 2 diabetes, since it is well-established that the translocation of GLUT4 to the membrane is the rate-limiting step in insulin-induced glucose sensitivity through activation of AKT in human patients, yet the detailed mechanisms remain elusive [51]. In addition, although the focus of the present study was on ROCK2 because of the elevation of its expression in hearts from diabetic rats and in vascular smooth muscle cells cultured in high glucose, in future it will also be important to determine whether ROCK1 contributes to diabetic cardiomyopathy. Others have reported increased ROCK1 expression in aorta from diabetic rats [52], and ROCK1 has been shown to play an important role in other forms of cardiac dysfunction, including heart failure [53].

In summary, the current study shows ROCK2 acting as a kinase for PKCβ2 T641 phosphorylation and activation in diabetic hearts. Inhibition of ROCK is associated with activation of PDK-1 and its downstream target AKT, leading to increased translocation of GLUT4 to the membrane. Overall, this study establishes that ROCK2 as a critical node in the development of diabetic cardiomyopathy and an important target for its treatment, as it’s inhibition will simultaneously improve multiple signaling pathways in the diabetic heart.
